# LiAlSiO_4_-coated Li_1.2_Mn_0.54_Ni_0.13_Co_0.13_O_2_ cathode: Enhancing Li-ion battery performance

**DOI:** 10.1371/journal.pone.0318327

**Published:** 2025-02-25

**Authors:** Shang-Mei Yang, Shi-Ping Shao, Yu-Long Xie

**Affiliations:** School of Chemistry and Chemical Engineering, Qinghai Minzu University, Key Laboratory of Resource Chemistry and Eco-environmental Protection in Tibetan Plateau of State Ethnic Affairs Commission, Qinghai Provincial Key Laboratory of Nanomaterials and Nanotechnology, Xining, China; Washington State University, UNITED STATES OF AMERICA

## Abstract

The lithium fast ion conductor LiAlSiO_4_ demonstrates exceptional lithium-ion transmission properties alongside remarkable chemical stability. Utilizing sol-gel techniques, we synthesized LiAlSiO_4_-coated cathode materials (LNCM@LASO) based on Li_1.2_Mn_0.54_Ni_0.13_Co_0.13_O_2_ to enhance their electrochemical performance. Rm space groups were identified in all materials through high-intensity diffraction peaks, indicating the presence of hexagonal layered α-NaFeO_2_ structures. Benefiting from the coating layer of LiAlSiO_4_, the conductivity and electrochemical performance of Li_1.2_Mn_0.54_Ni_0.13_Co_0.13_O_2_ are significantly improved. Compared with the unmodified LASO-0 sample (42.27%), the LASO-3 sample exhibits a superior initial coulomb efficiency of 66.02%. At various charge/discharge rates (0.1, 0.2, 0.5, 1, and 2 C), the LASO-3 electrode exhibits specific discharge capacities of 210.6, 189.3, 168.1, 151.8, and 125.2 mAh·g^−1^, correspondingly. Upon reverting the current density from 2 C to 0.1 C, the discharge capacity of the LASO-3 electrode rebounds to 206.4 mAh·g^−1^. After 100 cycles at 0.1 C, the LASO-3 electrode achieves a peak capacity retention rate of 88.9%. The superior conductive properties and chemical stability of the LNCM@LASO enhance the electron and ion transfer, thereby preventing electrolyte attack and boosting the electrochemical performance. This research marks a crucial step towards developing high-capacity, low-cost lithium-ion batteries with wide-ranging implications across multiple disciplines and industries.

## 1 Introduction

Layered oxide materials have become the dominant cathode materials since the commercialization of lithium-ion batteries in the 1990s, especially after the development of LiCoO_2_. However, the current layered oxides cannot meet the future energy storage requirements [1]. Lithium-rich manganese-based cathode material, xLi_2_MnO_3_·(1-x) LiMO_2_ (M = Ni, Co, Mn or/and Cr) (LNCM), is a promising cathode material first proposed by Numata [2] in 1997. Due to its high reversible specific capacity of more than 250 mAh·g^−1^, high operating voltage (>3.5 V. Li/Li^+^), low toxicity, and low economic cost. LNCM plays a key role in portable electronic devices and is also considered an ideal power solution for next-generation hybrid electric vehicles and electric cars [3,4]. Li_1.2_Mn_0.54_Ni_0.13_Co_0.13_O_2_ has attracted considerable research interest among these materials [5–7]. This material has a bilayer structure, characterized by two phases: the triangular LiMO_2_ phase with an Rm space group and the Li_2_MnO_3_ phase with a C2/m space group [8]. The activation of the Li_2_MnO_3_ phase activation in the charging process results in high capacity, while the LiMO_2_ phase primarily stabilizes the crystal structure. In the initial charge cycle, when the voltage is equal to or exceeds 4.5 V, the Li_2_MnO_3_ component is activated, this leads to the redox of transition metal ions and the production of a high capacity. However, this process irreversibly generates Li_2_O and MnO_2,_ releasing oxygen and causing a substantial irreversible capacity loss [9]. This loss of Li^+^ and O^2−^ and the reduction of lithium-ion sites within the lattice contribute to a low initial coulomb efficiency [10]. During cycling, unstable transition metal ions gradually occupy octahedral positions, reducing available positions for lithium ions and increasing voltage loss. In addition, the excess lithium ions occupying the position of non-electrochemically active tetrahedron form a spinel structure, further contributing to voltage attenuation of LNCM during cycling. Finally, the low electron conductivity of the Li_2_MnO_3_ component impairs the magnifying performance of LNCM [11].

To overcome the aforementioned challenges, researchers have proposed various modification strategies in recent years, including techniques like doping in bulk doping and surface coating [12–21]. Coating the surface serves as an effective approach to creating a physical barrier that prevents side reactions between electrode surface and electrolyte. This approach improves the surface durability of the coating material, enhances the transport efficiency of lithium-ion, and augments the electrical conductivity within the electrode laminate, thereby reducing material impedance. During subsequent charge/discharge cycles, a stable CEI film forms that greatly improves initial discharge capacity and cycling capacity by mitigating electrolyte decomposition and maintaining oxidation ion vacancies [22–24]. Therefore, surface coating has found widespread application in the modification of electroactive material surfaces. In recent research, a variety of materials for surface coating including Li_2_MnO_3_ [25], ZrO_2_ [26], AlF_3_-Al_2_O_2_ [27], Li_4_Ti_5_O_12_ [28], and others [29,30], have been employed to improve the electrochemical characteristics of LNCM. Another noteworthy coated material, LiAlSiO_4_, has effectively enhanced the electrochemical performance of diverse cathode materials including LiNi_0.5_Mn_1.5_O_4_ [31], Li(Li_0.17_Ni_0.2_Co_0.05_Mn_0.58_)O_2_ [32], LiNi_0.8_Co_0.15_Al_0.05_O_2_ [33], and LiNi_0.8_Mn_0.1_Co_0.1_O_2_ [34]. The remarkable lithium-ion conductivity and exceptional chemical stability are the main contributors to this enhancement. To our knowledge, there are no previous reports on utilizing LiAlSiO_4_ coating to improve the electrochemical performance of LNCM.

In this study, we employed a sol gel method to approach Li_1.2_Ni_0.13_Co_0.13_Mn_0.54_O_2_@LiAlSiO_4_ (LNCM@LASO) composite materials. Through a comprehensive series of physical and electrochemical characterizations, we thoroughly investigated the related properties of the composites.

## 2 Materials and methods

### 2.1 Preparation of both the pristine and LiAlSiO _4_ -coated material

The initial layered oxide LNCM powder was synthesized by the sol-gel method followed by high-temperature combustion, using a method similar to a previously reported [30]. To achieve this, CH_3_COOLi·2H_2_O, Ni(CH_3_COO)_2_·4H_2_O, Co(CH_3_COO)_2_·4H_2_O, and Mn(CH_3_COO)_2_·4H_2_O were dissolved in deionized water in a stoichiometric ratio of 1.26:0.13:0.13:0.54, yielding a clear and transparent solution. Subsequently, equimolar citric acid was dissolved in deionized water and slowly added drop by drop to the solution containing lithium and transition metals. The solution was vigorously stirred in a water bath at 90° C, resulting in the formation of a purple wet gel as the water evaporated. The gel was then dried in a blast drying an oven at 120° C for 12 h, resulting in a purple xerogel. After complete grinding, the material was transferred to a box furnace and subjected to heated at 480° C for 5 h, resulting in a black precursor. This precursor was further ground using a mortar and pestle, then sintered at 900° C for 12 h in the air to produce pristine LNCM.

For preparation of LNCM@LASO, we employed a citric acid-based sol-gel technique. The prepared LNCM powders were dispersed in an alcohol solution containing CH_3_COOLi·2H_2_O, AlNO_3_·9H_2_O, and (CH_3_CH_2_O)_4_Si. Subsequently, excess citric acid was introduced into the dispersion. Following stirring at 80° C for 4 hours, a viscous slurry was obtained, which underwent drying at 120° C and decomposition at 500° C for 6 hours in the air to form the LiAlSiO_4_ phase. The content of LiAlSiO_4_ coating ranged from 0 to 4 wt% of the LNCM powder, denoted as LASO-0, LASO-1, LASO-2 LASO-3, and LASO-4, respectively.

### 2.2 Materials characterization

The crystalline structure of LNCM and LiAlSiO_4_ was analyzed using powder X-ray diffraction (XRD), utilizing Cu-Kα radiation. Scanning was performed in the range of 10 to 90 degrees at a rate of 10 °·min^−1^. Scanning electron microscopy (SEM; GeminiSEM300) was used to visually observe the surface morphology of the sample. During the test, the sample was secured onto the metal sample table using conductive tape. An accelerated voltage of 5 kV was applied, enabling the rough determination of the sample particle size. For further analysis of morphology, particle size, and microstructure, Transmission electron microscopy (TEM; The JEM-2100, Japan) was employed. The TEM specifications include a point resolution of 0.23 nm, lattice resolution of 0.14 nm, acceleration voltage range of 80–200 kV, and magnification range of 50–1500000. X-ray Photoelectron Spectroscopy (XPS) utilizes X-ray radiation to excite and emit valence or inner electrons of atoms or molecules. Elements with atomic numbers equal to or greater than 3 can be detected using this technique. XPS has found a significant application in the characterization of cathode materials for lithium-ion batteries. Primarily used to measure element types and valence states on material surfaces. The X-ray photoelectron spectrometer Thermo Field ESCALAB 250Xi is used for testing in this paper. The excitation light source uses Al-Kα ray, the energy is 1486.6 eV, the working voltage is 14.6 kV, and the vacuum degree of the analysis chamber is 4 × 10^−9^ mbar. The standard is calibrated by C 1s = 284.8 eV.

### 2.3 Electrochemical measurement

Material electrochemical characterization was conducted through CR2032 coin cell tests. The cathode electrodes were fabricated using a composition comprising cathode powder, Super P, and PVDF (mass ratio of 8:1:1) dissolved in N-methyl-2-pyrrolidone (NMP). After thorough mixing, a 100 μm notch bar spreader was used to evenly spread the slurry onto aluminum foil. It was then underwent drying at 120° C for 12 h within a vacuum oven. The resulting electrode was then cut into 14 mm diameter circular plates after compression at 10 MPa for 1 minute. All CR2032 coin cells were assembled within an argon-filled glovebox (H_2_O, O_2_ < 0.1 ppm), employing lithium metal as the anode. LiPF_6_ (1 M) was dissolved in a mixture of ethylene carbonate and ethyl methyl carbonate (3:7 weight ratio) and a 25 μm trilayer polypropylene-polyethylene-polypropylene membrane (MTI) were used as the electrolyte and separator, respectively. The electrochemical performance was assessed utilizing a land battery testing system (LAND CT2001A). Charging and discharging of all cells were conducted at ambient temperature, within a potential range spanning from 2.0 to 4.8 V versus Li/Li^+^. The electrochemical workstation, CHI660B, facilitated the Electrochemical Impedance Spectroscopy (EIS) and cyclic voltammetry (CV) tests for the cells. EIS test parameters included an amplitude of 10 mV amplitude and a frequency range spanning from 10 MHz to 100 KHz. Meanwhile, CV parameters entailed a voltage range from 2.0 to 4.8 V and a scanning rate of 0.1 mV·s^−1^.

## 3 Result and discussion

### 3.1 Structures and discussion

**Fig 1:**
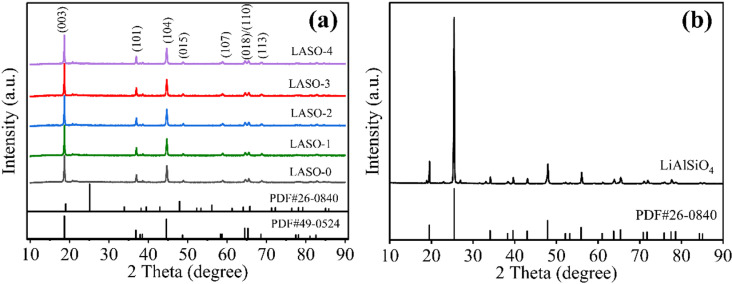
XRD spectra of (a) LASO-X (X = 0, 1, 2, 3, 4) and (b) LiAlSiO_4_.

**Fig 2:**
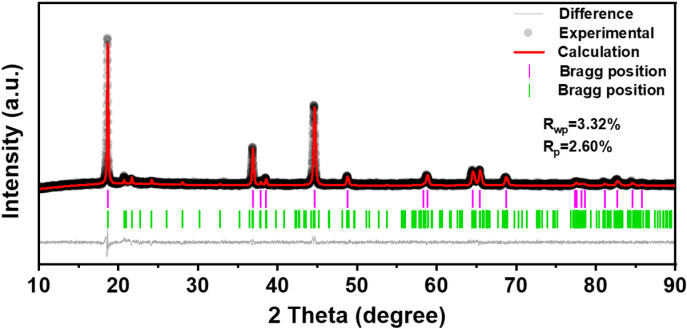
Rietveld refinement mapping of sample LASO-3.

Fig 1 shows the XRD signals of all the obtained LNCM samples LASO-X (X = 0, 1, 2, 3, 4) and LiAlSiO_4_ cathode materials. Fig 1a presents a comparison of XRD patterns of five samples of LASO-X (X = 0, 1, 2, 3, 4) [35]. All synthetic materials exhibit high-intensity diffraction peaks corresponding to hexagonal layered α-NaFeO_2_ structures with Rm space groups. The low-intensity diffraction peak between 20° and 25° indicates the monoclinic phase of Li_2_MnO_3_ with C2/m space group, affirming the formation of Li-rich materials due to the ordered arrangement of Li and Mn components in the LiMnO_2_ layer in a small range, forming a superlattice [36,37]. The results of Rietveld refinement of all samples using GSAS software are shown in Fig 2. The refinement results show that the Li_2_MnO_3_ content in LASO-3 reaches 28.3%, which confirms the presence of Li_2_MnO_3_. For the Li_1.2_Mn_0.54_Ni_0.13_Co_0.13_O_2_ cathode material, the characteristic peaks of various materials remain consistent before and after coating, this indicates that the presence of the lithium fast ion conductor LiAlSiO_4_ on the surface of the cathode material does not induce any structural changes in the raw materials. Notably, distinct crack peaks corresponding to (006)/(102) and (108)/(110) are observed, suggesting an optimal layered structure.

In addition, the c/a ratio serves as another method to evaluate the layered structure. The lattice parameters calculated through by the Jade software are presented in Table 1. In this study, the c/a value inside the lattice of both LNCM and LASO-X (X = 0, 1, 2, 3, 4) are exceed 4.9, indicating that the material is a cubic close-packed structure. A larger c/a value indicates an increased internal volume within the material, which in this case, signifies a wider lithium-ion de-intercalation channel [35,38]. The I(003)/I(104) ratio indicates the degree of Li-Ni ion mixing within the layered hexagonal structure. Generally, a higher I(003)/I(104) value indicates a lower degree of cation mixing [39]. As the coating amount increases, there is a gradual rise in the I(003)/I(104) value, accompanied by a gradual decrease in the degree of cation mixing. However, when LASO-4 is reached, the I(003)/I(104) value starts to decrease. The XRD pattern does not exhibit prominent peaks corresponding to Si and Al elements. This observation may be due to the relatively low coating amount and the low elemental content. To validate the synthesis of LiAlSiO_4_ phase on the surface of LNCM, we analyzed the pure coating material synthesized under the same conditions was analyzed by XRD, the results are depicted in Fig 1b. Upon compared with the LiAlSiO_4_ standard PDF card in the database, most peaks of the synthesized LiAlSiO_4_ phase closely match the standard, validating the formation of LiAlSiO_4_ under the synthetic conditions.

**Table 1:** Lattice parameters of LASO-X (X = 0, 1, 2, 3, 4).

Sample	a/(nm).	c/(nm).	c/a	I_(003)_/I_(104)_
LASO-0	2.8556	14.2605	4.9939	1.5267
LASO-1	2.8545	14.2262	4.9837	1.8116
LASO-2	2.8596	14.2394	4.9794	1.7636
LASO-3	2.8590	14.2526	4.9852	1.9011
LASO-4	2.8598	14.2270	4.9748	1.8215

**Fig 3:**
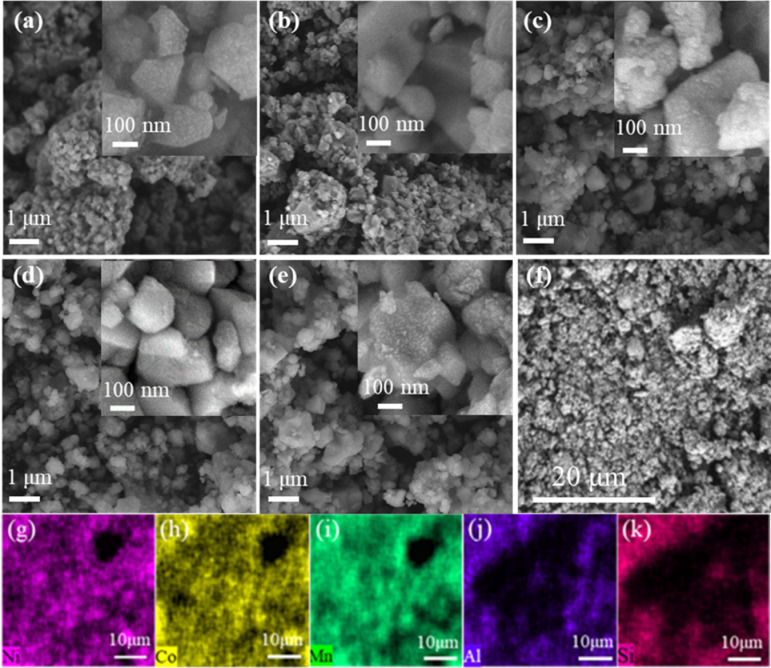
(a–e) SEM images of LASO-X (X = 0, 1, 2, 3, 4); (f–k) Elemental mapping of LASO-3 via EDS.

We examined the morphologies of these materials via SEM, as visualized in Fig 3 From Figs 2e and 3a, all sample morphologies exhibit small irregular particles without any noticeable aggregation, and the formation of these uniformly sized particles can be attributed to the use of the chelating agent, citric acid [40]. In addition, the presence of LiAlSiO4 has minimal effect on particle growth, as the morphology of the LNCM remains unchanged [33]. Furthermore, the particle dispersion of the samples gradually improves from LASO-0 to LASO-3, with LASO-3 demonstrating the most uniform particle dispersion size. In particular, smaller particle sizes enhance electrochemical performance. LASO-4 exhibits slight agglomeration, potentially due to the excessive thick coating layer resulting in a rough material surface. The EDS mapping image of LASO-3 at 20 μm is presented in Figs 3f–3k, showcasing the distribution of Ni, Co, Mn, Al, and Si, while Li remain undetectable due to its low atomic mass [40].

The surface of LNCM exhibits even distribution of all elements. Table 2 sequentially presents the element distribution of O, Mn, Co, Ni, Al, and Si, confirming the uniform coating of LiAlSiO_4_ on the surface of the Li-rich manganese-based cathode material [41].

**Table 2:** The element content rates of O, Mn, Co, Ni, Al, and Si as per the EDS mapping results.

Element	Mass (%)	Atom (%)
O	22.80	50.07
Mn	49.42	31.60
Co	12.82	7.64
Ni	12.43	7.44
Al	1.78	2.31
Si	0.75	0.94
Total	100.00	100.00

**Fig 4:**
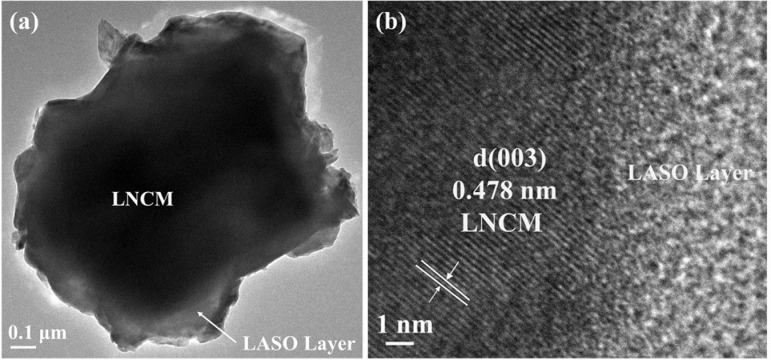
(a) TEM image of LASO-3; (b) HRTEM image with the inset showing the (003) plane.

The TEM image (Fig 4a) for the LASO-3 sample clearly shows that the compound has clear boundaries comprising dark cathode material in the middle and a delicate coating layer on the surface. Fig 4a displays homogeneous growth of the LASO nanoparticles on the LNCM surface, consistent with the SEM image. The lattice-resolved HRTEM of the LASO-3 sample in Fig 4b shows a spacing of 0.478 nm, which aligns with the (003) plane of the LNCM phase, supporting the results of the XRD analysis [39,42].

**Fig 5:**
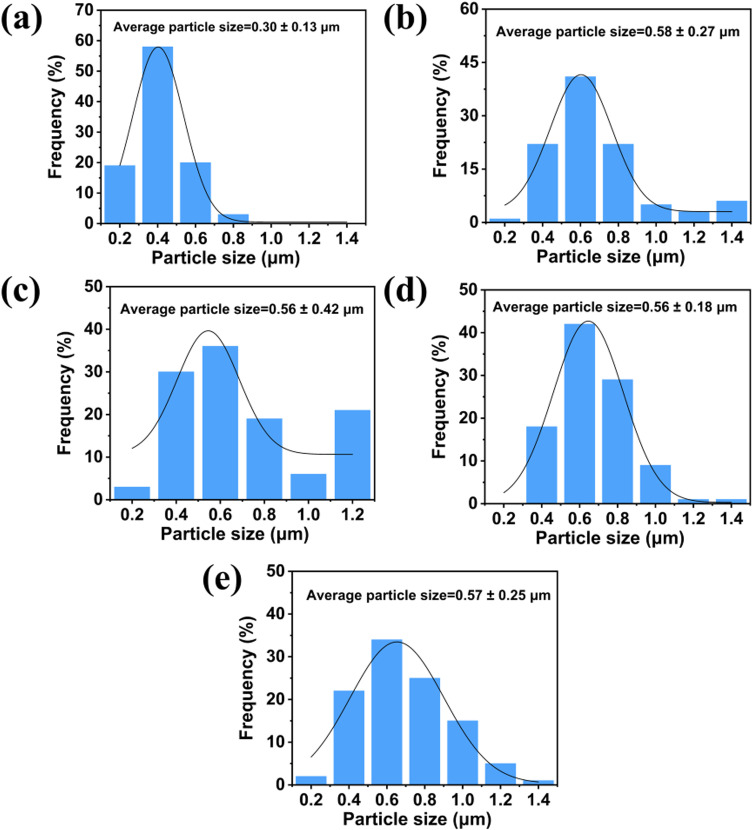
Particle size distribution of LASO-X (X= 0, 1, 2, 3, 4).

To further explore the relationship between particle size and material properties, particle size analysis of materials was performed. From SEM images of the same magnification, 100 groups of particles were selected for each material. As shown in Fig 5, the particle size distribution of the material primarily ranges from 0.1–1.5 μm. The average particle size of the uncoated positive electrode material LNCM in Fig 5a is about 0.3 μm, the particle size of the coated material increases after modification. Figs 4e and 5b shows that as the quantity of LiAlSiO_4_ coating increases, the average particle size of the modified material remains between 0.56 and 0.58 μm. Additionally, the LASO-3 material’s normal distribution curve of the LASO-3 material shows the lowest degree of dispersion. When sample particles are evenly dispersed, metal ions can stably participate in the electrochemical reaction, effectively reducing electrode polarization and facilitating rapid lithium ion migration [43–45].

**Fig 6:**
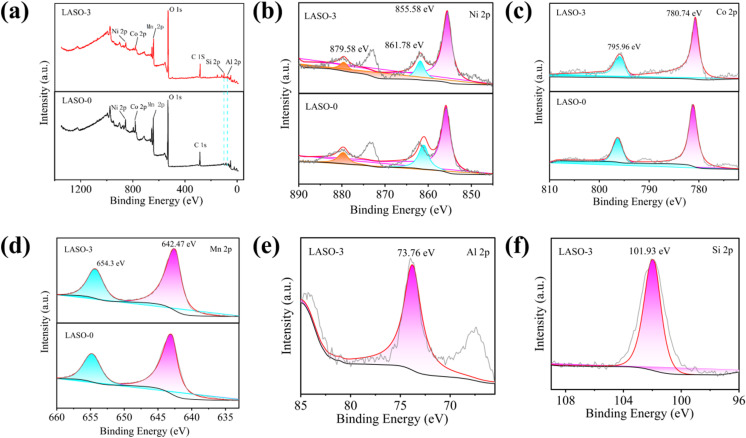
Comparison of XPS spectra of LASO-0 and LASO-3 (a) full spectrum; (b, d) XPS spectrum of Ni 2p, Co 2p, Mn 2p; (e) Si 2p spectrum; (f) Al 2p spectrum.

To determine the elemental valence distribution in LiAlSiO_4_-coated Li-rich manganese-based cathode materials, XPS tests were carried out on LASO-3 materials, and the binding energy of the measured elements was calibrated using an external carbon source C 1s (284.8 eV). In the XPS spectrum, Ni 2p, Co 2p and Mn 2p split into two peaks corresponding to different valence states for nickel, cobalt and manganese. In general, the lower energy 2p_3/2_ main peak corresponds to the valence state of the element, while the 2p_1/2_ peak provides additional information about the valence state. By analyzing the position and relative strength of these peaks, the chemical state of the elements within the compound can be inferred [46–48]. Fig 6a shows the full spectrum comparison of elements in LASO-0 and LASO-3 materials, and Figs 6b–6f shows the fine spectrum comparison of Ni 2p, Co 2p, Mn 2p, Si 2p and Al 2p in the two materials respectively. The spectra of the material before and after modification are basically the same, and it can be seen from the full spectrum comparison that LASO-3 contains obvious Si 2p and Al 2p peaks. The main peak of the binding energy of Ni 2p_3/2_ orbit is 855.58 eV, and the satellite peak is 861.78 eV. The peak of the binding energy of the Ni 2p_1/2_ orbital binding energy is 872.7 eV, the satellite peak is 879.58 eV, and the energy level difference of the splitting Δ*E* = 17.12 eV, indicating that the Ni elements in the sample before and after the coating modification are + 2 valence. Analysis of the 2p orbital splitting peak binding energy of Co shows that the orbital binding energy of Co 2p_3/2_ is 780.74 eV, while that of Co 2p_1/2_ is 795.96 eV, and the energy level difference of the splitting Δ*E* = 15.22 eV, indicating that all the Co elements in the sample are + 3 valence. From Fig 6d, it is evident that the Mn 2p orbit presents two primary peaks: Mn 2p_3/2_ at 642.47 eV and Mn 2p_1/2_ at 654.3 eV. There is no corresponding satellite peak appears, and the binding energy position corresponds to Mn^4+^. The orbital binding energy of Si 2p_3/2_ is 101.93 eV, the binding energy position corresponds to Si^4+^, the orbital binding energy of Al 2p_3/2_ is 73.76 eV, the binding energy position corresponds to Al^3+^, and in the fast ion conductor LiAlSiO_4_, the element valence state of Al is usually +3. Since aluminum typically loses three electrons to form the Al^3+^ ion, the elemental valence state of Si is typically +4, because silicon typically loses or shares four electrons to form the Si^4+^ ion. XPS analysis indicates that Ni, Co and Mn are +2 valence, +3 valence and +4 valence in Li-rich manganese-based cathode materials both before and after LiAlSiO_4_ coating.

### 3.2 Electrochemical performance

**Fig 7:**
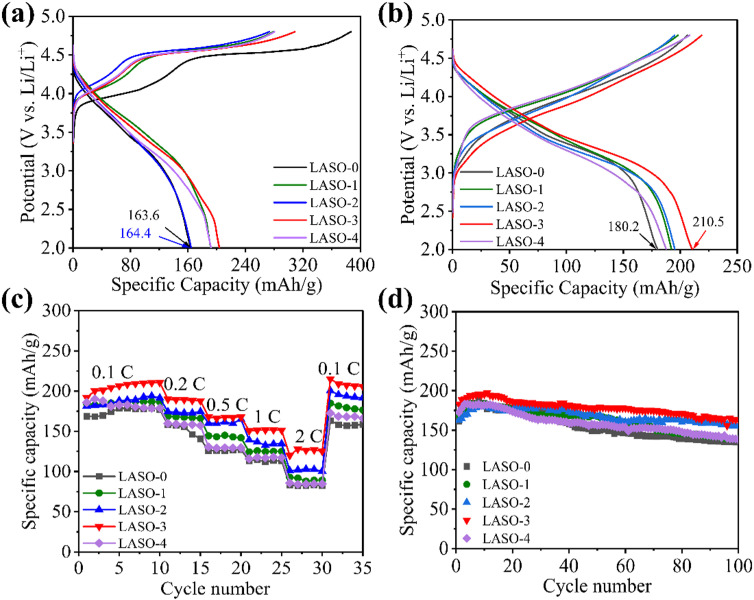
(a) Initial charge–discharge curves, (b) charge–discharge curves after 10 cycles, (c) rate performance curves, and (d) cyclic curves after 100 times at 0.1 C of LASO-X (X = 0, 1, 2, 3, 4).

Fig 7a plots the first charge and discharge curves of LNCM (LASO-0) and LASO-coated (LASO-1, LASO-2, LASO-3, and LASO-4) materials. Measurements were performed at ambient temperature (25°C), within a voltage range of 2.0–4.8 V, and at a current density of 0.1 C. Overall, the electrochemical properties demonstrated by all of the cells are in agreement with those reported in the literature. Furthermore, no novel profiles of the surface-coated electrodes are observed, suggesting that the surface coating is not involved in the electrochemical process. Two voltage platforms are present in the initial charge curve, where the inclined region below 4.5 V corresponds to the migration of Li^+^ and the oxidation of low-priced transition metals within the LiTMO_2_ components (Co^3+^ oxidation to Co^4+^ and Ni^2+^ oxidation to Ni^4+^). The platforms above 4.5 V belong to the activation of Li_2_MnO_3_ components [43]. During the activation process of Li_2_MnO_3_ in the platform region, there is excessive oxidation of lattice oxygen (in the form of O_2_ or Li_2_O), Li^+^ are inserted into the material lattice during charging but cannot return during discharge, leading to a decrease in specific discharge capacity [49,50]. Upon comparing the first charge and discharge curves of the five samples, it is observed that the initial coulomb efficiency of the modified LASO-3 sample (66.02%) surpasses that of the unmodified LASO-0 sample (42.27%). However, the overall coulombic efficiency of the first lap before and after modification is low, which is mainly due to the irreversible phase changes and side reactions that occur in the cathode material during the first charge/discharge process. These side reactions lead to partial capacity irreversibility. As well as the fact that the cathode material and the electrolyte are in a break-in stage in the first lap also contributes to the low Cullen efficiency in the first lap. This is attributed to the effective reduction of surface Li_2_O/LiOH/Li_2_CO_3_ and other lithium residues by the LiAlSiO_4_ layer on the cathode surface [33]. Subsequently, after several charge and discharge cycles at 0.1 C, the charge and discharge curve of the battery was analyzed when the Coulomb efficiency tended to be stable, as shown in Fig 7b. The figure reveals the appearance of a tiny platform at the end of the discharge process (around 2.7 V), corresponding to the reduction of transition metal ions. Within the charge/discharge curve, the specific discharge capacity of the material gradually increases with the coating layer, from 180.2 mAh·g^−1^ for LASO-0 to 210.5 mAh·g^−1^ for LASO-3. It was shown that the coating effectively isolated the cathode material from the electrolyte, thus inhibiting HF corrosion. In particular, the lithium fast ion conductor LiAlSiO_4_ provides high ionic conductivity and reduces the resistance of interfacial charge transfer resistance, enabling high capacity release. As the coating layer grows to a certain thickness, the specific discharge capacity decreases due to the inability of LiAlSiO_4_’s incapacity to provide lithium storage, and thicker coating leads to the growth of Li^+^ diffusion paths, which hinders Li^+^ insertion and extraction.

Fig 7c presents the discharge performance of the five materials under different current densities, revealing that the superior discharge performance of LASO-3 compared to the other electrodes. The LASO-3 electrode exhibits specific discharge capacities of 210.6, 189.3, 168.1, 151.8, and 125.2 mAh·g^−1^ under charge/discharge rates of 0.1, 0.2, 0.5, 1, and 2 C, respectively. As the current density reverts from 2 C to 0.1 C, the discharge capacity of LASO-3 electrode is recovered, and the specific discharge capacity is restored to 206.4 mAh·g^−1^. In addition, after undergoing 100 cycles at 0.1 C, the capacity retention rate peaks at 88.9% after 100 cycles at 0.1 C, as shown in Fig 7d. Table 3 presents a comparison of the capacity retention rates of various cathode materials following 100 cycles. The results indicate that the existence of LiAlSiO_4_ coating can effectively prevent the occurrence of side reactions and stabilize the electrode surface structure [51].

**Table 3:** The capacity retention of LASO-X (X = 0, 1, 2, 3, 4) after 100 cycles at 0.1 C.

Cathode material	Capacity retention (%)
LASO-0	82.14
LASO-1	83.78
LASO-2	88.90
LASO-3	94.76
LASO-4	80.66

**Fig 8:**
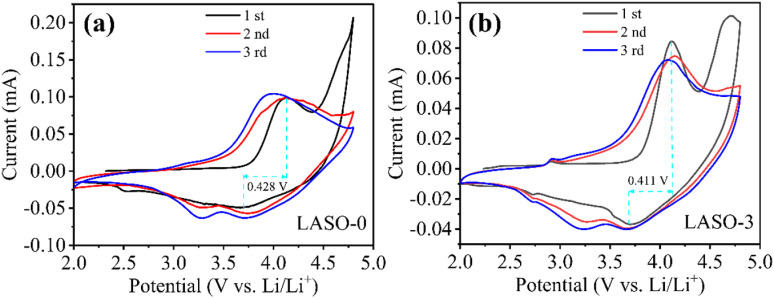
CV curves for the initial three cycles of (a) LASO-0 and (b) LASO-3.

The cyclic voltammetry (CV) curves of the pure phase (Fig 8a) and coated modification (Fig 8b) are shown in Fig 8. CV testing was performed with a scan voltage range of 2.0 to 4.8 V and a scan rate of 0.1 mV·s^−1^. During the initial cycle, the oxidation peak at approximately 4.1 V signifies the extraction of Li^+^ and the oxidation of Ni^2+^ and Co^3+^ as part of the charging process in the Li-rich manganese-based material [52]. Meanwhile, the oxidation peak around 4.7 V corresponds to the activation of the Li_2_MnO_3_ phase and the conversion of Li_2_O to MnO_2_. During the discharge process, the reduction peak at approximately 3.62 V indicates the insertion of Li^+^ into the layered lattice. In the first charge and discharge, the redox peak is notably shifted compared to the second and third cycles. The apparent shift of the redox peak is due to the side reaction between the positive electrode material and the electrolyte as the number of cycle turns increases. Insoluble products are generated, which may change the properties of the electrode surface, which in turn affects the charge transfer rate and current density, leading to a shift in the redox peak potential. Irreversible products may also be generated, resulting in an irreversible reaction [53]. However, the curves for the 2nd and 3rd cycles exhibit a high degree of overlap, showing the best agreement. In addition, the decrease in intensity or shift in peak potential in the second and third cycles also indicates an irreversible phase transition within the material, leading to a loss of capacity. The aforementioned results are consistent with the internal mechanism and typical charging and discharging characteristics of lithium-rich manganese-based materials. As demonstrated in Fig 8, the peak current of the redox peak in LASO-3 material is significantly higher than that in LASO-0 material, and the redox peak potential difference of LASO-3 (0.411 V) is obviously smaller than that of LASO-0 (0.428 V), indicating a superior dynamic performance of LASO-3 compared to LASO-0.

**Fig 9:**
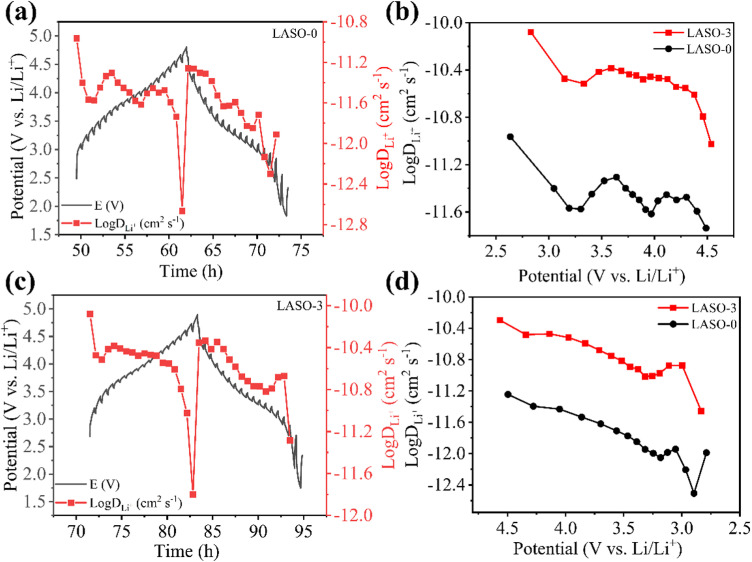
(a, c) GITT test curve and lithium ion diffusion curve of LASO-0 and LASO-3 materials, respectively, (b) LogDLi+~E diagram of LASO-0 and LASO-3 materials under charging, and (d) LogDLi+~E diagram for the determination of LASO-0 and LASO-3 materials under discharging.

In order to characterize the lithium storage process of the battery material, a constant current intermittent titration (GITT) technique was used. This technique is used to test the change of lithium diffusion state between different voltage intervals. In the GITT test, the LASO-0 and LASO-3 materials were charged for 30 min at a current density of 0.1 C within the voltage range of 2.0–4.8 V, and battery voltage was allowed to relax to a stable value in the open circuit voltage state for 40 min. This process was repeated until a high cut-off voltage (4.8 V) was reached, and the same discharge process was continued until a low cut-off voltage (2 V) was reached. Figs 9a and 9c reflects the diffusion of lithium ions throughout a complete charge and discharge process of LASO-0 and LASO-3 materials, respectively, and the variation trend of lithium-ion diffusion is identical. In combination with Figs 9b and 9d, the LogD_Li +_ value of the LASO-3 material at different voltages is higher than that of the LASO-0 material under both charging and discharging stages. This observation further proves that the LASO-3 material exhibits a higher electronic/ionic conductivity and better interface stability, thereby demonstrating excellent electrochemical performance.

**Fig 10:**
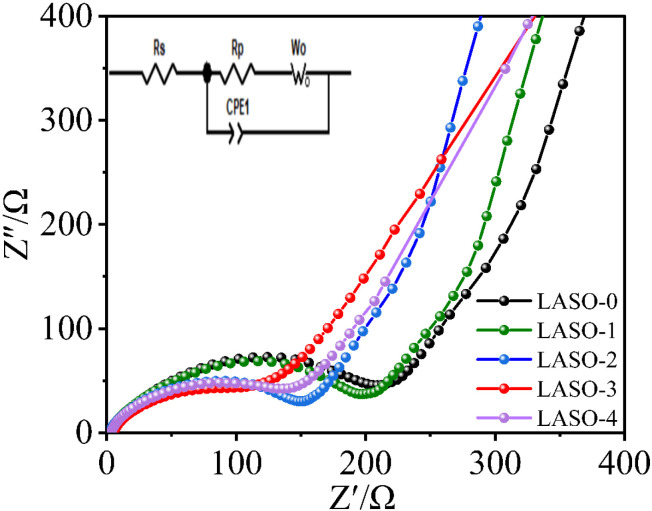
AC impedance spectra of the LASO-0 and LASO-3 materials.

Fig 10 illustrates the AC impedance plots of all the materials. The AC impedance curve consists of two parts: A semicircle in the high-frequency region and a straight line in the low frequency region. These sections represent the electrochemical charge transfer impedance at the electrode/electrolyte interface and the diffusion impedance of lithium ions in the solid phase, respectively. It shows that the lithium ion removal and insertion orbitals are wider and the lithium ion removal and insertion rate is faster in the LASO-3 material. The surface coating reduces the direct contact between the cathode material and the electrolyte, lowers the interfacial film resistance and enhances Li^+^ diffusion, resulting in a more stable surface structure.

**Fig 11:**
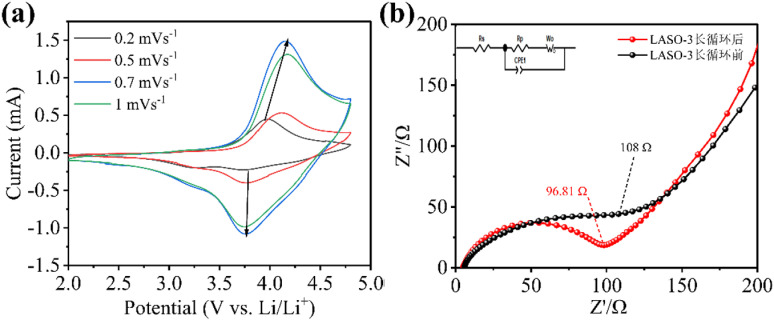
(a) Cyclic voltammetry results at different sweep speeds; (b) Ac impedance spectrum after 100 cycles at 0.1 C of LASO-3.

To further investigate the ion dynamics process inside the LASO-3 material, cyclic voltammetry tests were performed on the LASO-3 material at the scanning rates of 0.2, 0.5, 0.7 and 1 mV·s^−1^, and the results are shown in Fig 11a. With the increase of current density, it can be seen that the displacement of oxidation and reduction peaks of LASO-3 is small and can maintain good peak shape. It indicates that LASO-3 has a small electrochemical polarization problem and good electrochemical reversibility. It is also concluded that the lithium ion migration and electron transfer rate are well matched, which is the basis for capacity maintenance during long cycling. Fig 11b illustrates the AC impedance curve obtained from LASO-3 electrode material following 100 cycles of constant current charging and discharging at a rate of 0.1 C. After cycling, the charge transfer impedance notably decreases compared to pre-cycling values (from 127 Ω before cycling to 96.81 Ω after cycling). The decline in charge transfer impedance may be due to the coating of lithium fast ion conductor on the surface of the electrode material. A sequence of chemical reactions occur during the battery cycle, and the electrolyte interface forms SEI film, the SEI film is also a lithium ion conductor, which accelerates the rate of lithium ion removal and mitigates the charge transfer impedance at the interface.

## 4 Conclusions

In summary, LNCM@LASO composites were successfully prepared by the sol-gel method. SEM, TEM and EDS analyses revealed that the resultant LiAlSiO_4_ layer was well deposited on surface of LNCM. In particular, the remarkable increase rise in the specific discharge capacity of LASO-3 was attributed to the excellent lithium ion conductivity of the fast ion conductor LiAlSiO_4_, which accelerated the rate of lithium ion deposition at the material surface. The excellent electrochemical performance of the coated material was due to the LiAlSiO_4_ coating layer, which effectively isolated the cathode material from the electrolyte and mitigated side reactions, thereby stabilizing the surface structure. Our results show that LNCM@LASO is a promising material to be used as a cathode material for lithium-ion batteries. Overall, the proposed LiAlSiO_4_ cladding material study has injected new vitality into lithium anode materials. Based on this study, with further breakthroughs in materials science and preparation technology, the cladding performance of lithium cathode materials will be further improved. In turn, it will promote the development of the market.
